# Expression Signature of IFN/STAT1 Signaling Genes Predicts Poor Survival Outcome in Glioblastoma Multiforme in a Subtype-Specific Manner

**DOI:** 10.1371/journal.pone.0029653

**Published:** 2012-01-05

**Authors:** Christine W. Duarte, Christopher D. Willey, Degui Zhi, Xiangqin Cui, Jacqueline J. Harris, Laura Kelly Vaughan, Tapan Mehta, Raymond O. McCubrey, Nikolai N. Khodarev, Ralph R. Weichselbaum, G. Yancey Gillespie

**Affiliations:** 1 Department of Biostatistics (Section on Statistical Genetics), University of Alabama at Birmingham, Birmingham, Alabama, United States of America; 2 Department of Radiation Oncology, University of Alabama at Birmingham, Birmingham, Alabama, United States of America; 3 Department of Surgery (Neurosurgery), University of Alabama at Birmingham, Birmingham, Alabama, United States of America; 4 Department of Radiation & Cellular Oncology, Ludwig Center for Metastasis Research, University of Chicago, Chicago, Illinois, United States of America; University of Chicago, United States of America

## Abstract

Previous reports have implicated an induction of genes in IFN/STAT1 (Interferon/STAT1) signaling in radiation resistant and prosurvival tumor phenotypes in a number of cancer cell lines, and we have hypothesized that upregulation of these genes may be predictive of poor survival outcome and/or treatment response in Glioblastoma Multiforme (GBM) patients. We have developed a list of 8 genes related to IFN/STAT1 that we hypothesize to be predictive of poor survival in GBM patients. Our working hypothesis that over-expression of this gene signature predicts poor survival outcome in GBM patients was confirmed, and in addition, it was demonstrated that the survival model was highly subtype-dependent, with strong dependence in the Proneural subtype and no detected dependence in the Classical and Mesenchymal subtypes. We developed a specific multi-gene survival model for the Proneural subtype in the TCGA (the Cancer Genome Atlas) discovery set which we have validated in the TCGA validation set. In addition, we have performed network analysis in the form of Bayesian Network discovery and Ingenuity Pathway Analysis to further dissect the underlying biology of this gene signature in the etiology of GBM. We theorize that the strong predictive value of the IFN/STAT1 gene signature in the Proneural subtype may be due to chemotherapy and/or radiation resistance induced through prolonged constitutive signaling of these genes during the course of the illness. The results of this study have implications both for better prediction models for survival outcome in GBM and for improved understanding of the underlying subtype-specific molecular mechanisms for GBM tumor progression and treatment response.

## Introduction

Glioblastoma multiforme (GBM) remains the most common primary brain malignancy and carries the worst prognosis [1]. In recent years, several groups have investigated molecular and genetic characteristics of these tumors in order to develop both prognostic and predictive biomarkers. Most of the biomarkers identified to date have been prognostic in that they help to determine estimates of survival (prognosis) independent of treatment. Predictive markers, on the other hand, inform regarding sensitivity to specific therapies. Predictive markers in GBM are quite limited, with the only established marker being the methylation status of O(6)-methylguanine-DNA-methyltransferase (MGMT) which is a predictor of temozolomide [2] and radiation resistance [3]. However, studies from other cancers have identified predictive markers with potential application in GBM.

Signal transducer and activator of transcription 1 (STAT1), the putative downstream effector of interferon (IFN), and interferon-related genes have been identified as key regulators of radiation resistance in preclinical models of head and neck squamous cell cancer [4], [5] and have been identified as radiation inducible in a wide variety of cancer cell lines, including glioma [5], [6]. Moreover, IFN/STAT1 signaling has been associated with not only metastatic potential, but also resistance to adriamycin chemotherapy and radiation in a murine model of melanoma [7]. Importantly, these results have been confirmed in breast cancer patients in which an “IFN-related DNA damage resistance signature” (IRDS) provided an improved outcome classification in terms of locoregional failure following adjuvant radiation and efficacy of adjuvant chemotherapy [8]. Because of the results of these experimental studies, and the observation that the IRDS gene expression pattern is also seen in high grade glioma primary tumors [8], we have hypothesized that up-regulation of these genes in GBM patients may be predictive of poor survival outcome and/or treatment response. To test this hypothesis, we have utilized gene expression data and clinical data from the Cancer Genome Atlas Project (http://cancergenome.nih.gov/) to test the association between an IFN/STAT1 pathway signature derived from the IRDS with survival outcome of GBM patients.

We have constructed an 8 gene set associated with the IFN/STAT1 pathway: STAT1, IFI44, IFIT3, OAS1, IFIT1, ISG15, MX1, and USP18 [8]. Survival analysis as a function of gene expression data was performed used Cox Proportional Hazards models. We have created single gene models, and we have created multiple gene models with various model selection techniques. In addition, previous reports indicate the presence of molecular subtypes of GBM (Classical, Mesenchymal, Proneural, and Neural [9]) which show distinct clinical and molecular characteristics. Thus we have also performed subtype-specific survival analysis to test whether survival outcome of GBM due to IFN/STAT1 genes is subtype-specific.

## Results

### Single Gene Models

**Figure 1:**
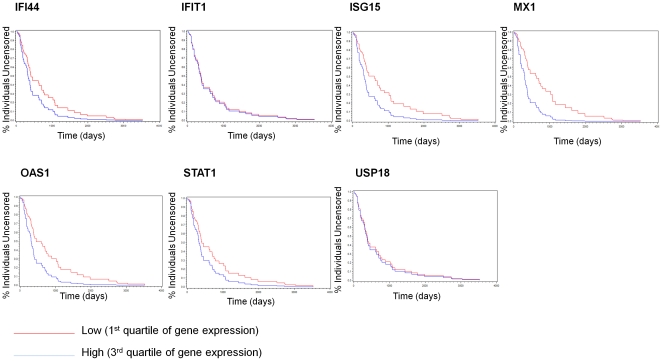
Survival Curves for age-adjusted Cox Proportional Hazard models for 1^st^ quartile (red) and 3^rd^ quartile (blue) gene expression values for each gene in the Proneural subtype.

Single gene Cox models were built with age as a covariate for each of the genes in the hypothesized signature except for IFIT3 which was missing from the gene-averaged expression data set. Table 1 shows the resulting models in the full data set (all samples) and the Proneural data set. The results for the other three subtypes are in Table S1. These results show an increased hazard for death for all of these genes in both the full and Proneural data sets, with significance at a level of 0.05 found for MX1 in the full data set, and significance found for all genes *except* IFIT1 and USP18 in the Proneural data set. The full and Proneural models are concordant, with MX1 showing the strongest effect in both, and a similar relative ranking of gene effects in each. The single gene results were not significant in the other subgroups tested (with the exception of the Neural group which had a significant effect for USP18, HR = 1.75, *p* = 0.02). In order to visualize the survival effects for the various single gene models, predicted survival curves for individuals at the 3^rd^ quartile (75%) and 1^st^ quartile (25%) of the expression distribution for each gene were graphed in Figure 1 for the Proneural model and in Figure S1 for the full model. The survival curves were generated using the median age, see details in the Methods section.

**Table 1:** Single Gene Cox Proportional Hazards Models with age adjustment for seven genes available in the TCGA discovery (gene-averaged) data set.

Gene	All		Proneural	
	*HR*	*p value*	*HR*	*p value*
**IFI44**	1.14 (0.98,1.33)	0.089	**1.55 (1.16,2.06)**	**0.003**
**IFIT1**	1.03 (0.92,1.16)	0.579	1.04 (0.80,1.33)	0.789
**ISG15**	1.14 (0.99,1.30)	0.060	**1.50 (1.14,1.96)**	**0.004**
**MX1**	**1.15 (1.01,1.30)**	**0.034**	**1.86 (1.37,2.52)**	**<.0001**
**OAS1**	1.14 (0.97,1.33)	0.097	**1.70 (1.19,2.41)**	**0.003**
**STAT1**	1.14 (0.94,1.37)	0.170	**1.62 (1.11,2.35)**	**0.011**
**USP18**	1.07 (0.87,1.31)	0.513	1.09 (0.69,1.70)	0.710

Estimated hazard ratios (95% confidence interval in parentheses) and p-values are given for each gene, and significant effects are shown in boldface.

### Multiple Gene Models

Because the genes in the STAT1/IFN signature are highly correlated (see correlation among genes in the full and Proneural data sets in Tables S2 and S3) and functionally related, we expected that a multi-gene model may take into account the complexities of the joint effects on survival of this signature. We used various methods for creating multi-gene models including stepwise regression with and without age adjustment, and elastic net which estimates a joint multi-gene model with a penalty for larger coefficients and more complex models. The results are shown in Table 2 which shows analysis in the full data set (all samples) and analysis in each of the four subtypes.

**Table 2:** Multi-gene Cox Proportional Hazards models for all samples and specific subtypes using three different model selection methods: Stepwise regression with age as a covariate (SW with age), stepwise regression without age (SW no age), and Elastic Net.

			HR (LB,UB)	
Model	Term	*SW with age*	*SW no age*	*Elastic Net*
**All**	**Age**	1.03 (1.02,1.04)	*NI*	1.02
	**MX1**	1.29 (1.06,1.57)	1.44 (1.19,1.75)	1.09
	**IFIT1**	0.88 (0.74,1.04)	0.79 (0.67,0.94)	*NI*
	**R^2^**	19%	7%	31%
**Proneural**	**Age**	1.04 (1.02,1.06)	*NI*	1.03
	**MX1**	2.12 (1.13,3.97)	2.44 (1.48,4.03)	1.88
	**IFIT1**	0.42 (0.23,0.76)	0.6 (0.4,0.92)	0.64
	**IFI44**	1.82 (0.97,3.41)	2.3 (1.28,4.15)	1.5
	**USP18**	0.46 (0.23,0.95)	0.36 (0.19,0.68)	0.6
	**ISG15**	1.93 (0.77,4.81)	*NI*	1.27
	**OAS1**	*NI*	*NI*	1.02
	**R^2^**	61%	47%	82%
**Neural**	**Age**	1.06 (1.01,1.1)	*NI*	1.03
	**USP18**	4.06 (1.47,11.19)	1.65 (1.04,2.62)	1.3
	**MX1**	0.48 (0.22,1.05)	*NI*	*NI*
	**IFIT1**	*NI*	*NI*	1.06
	**IFI44**	*NI*	*NI*	1.05
	**R^2^**	37%	14%	44%
**Classical**	**Age**	1.01 (0.99,1.04)		
	**R^2^**	3%		
**Mesenchymal**	**Age**	1.05 (1.02,1.08)		1.03
	**R^2^**	15%		24%

Hazard ratios with confidence limits are given for each term added to each model. If the term was not added to a given model, NI is displayed for “not included”. The total explained variance for each model (R^2^) is also displayed.

The first observation is that the multi-gene models confirm the single-gene results which show that the effect of this gene signature is most pronounced in the Proneural subgroup. In addition, the MX1 gene shows the largest effect in both the full and Proneural models (as it did in the single gene analysis). Of note is the fact that IFIT1 and USP18 are both added to the Proneural model for all three model selection methods (and IFIT1 is also added to the full model for the stepwise methods); these genes show non-significant effects in the single-gene analysis, but in the multi-gene analysis show effects in the opposite direction (high expression increases survival). Thus the multi-gene analysis shows that IFIT1 and USP18 may additionally be involved in survival prediction, even though they were not significant in the single-gene analysis. The inclusion of these genes in the multi-gene model is probably due to IFIT1 and USP18 having effects in the opposite direction (protective) after accounting for the main effect of MX1, a complex phenomenon that can only be captured with a multi-gene model.

**Figure 2:**
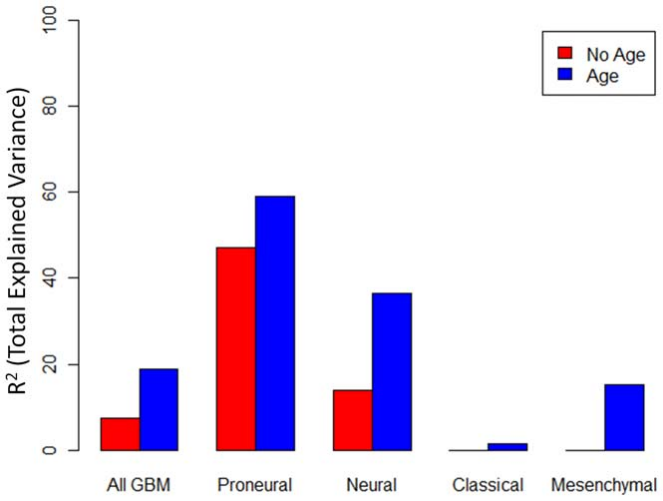
Bar plot of total explained variance (R^2^) for survival models discovered using stepwise selection with genes only (“No Age”) or with genes and age (“Age”) for all GBM patients and by subtype.

The elastic net results for the Proneural model show that a similar gene set is selected (as compared to the stepwise results), thus showing consistency between these two model selection techniques; however, more genes are added in the elastic net model with a higher R^2^ (82.1% as compared with 59.1% in the stepwise model). Another observation is that the models with and without age are similar, with the same genes added and with the same effect direction. This result indicates that the gene signature proposed here gives predictive value above and beyond age, the most commonly-used risk predictor for GBM, and furthermore, the effects on survival for these genes are largely independent of age. Another observation is that the models for the Neural and Proneural subtypes are quite different, with effects in the opposite direction for several genes, illustrating that the effect of this gene signature on survival is highly subtype-dependent. Figure 2 summarizes the results of the stepwise models in terms of total explained variance (R^2^) with and without age for the full analysis and analysis in each subgroup.

A test of the proportional hazards assumption of the Cox model, which requires that the hazard ratio for a term in the model be constant in time, was Tables S4 and S5, respectively). These tests showed that age showed a significant violation of the proportional hazards assumption, but none of the genes showed significant violations. To account for the proportional hazards violation for age, we examined the models without age, which showed no such violation (See Table S9 and Table 2, column 2). The full model and Proneural model showed the same genes added to the model with effects in the same direction and of similar size. Thus we conclude that the predictive value of these multi-gene models is present even when removing age which shows a proportional hazards violation.

### Probe Set Gene Expression Analysis

In order to verify that the survival models built here are valid in multiple gene expression platforms and not dependent on the specific algorithm used to build a gene-averaged, cross-platform gene signature [9], we also built survival models that included probe set expression data from two of the gene expression platforms in the TCGA project (Affymetrix and Agilent). Elastic net was used to build multi-gene models from the probe sets assigned to the eight genes in our signature along with age, and the resulting hazard ratios and model R^2^ values are shown in Table S6 for the Affymetrix probe sets and Table S7 for the Agilent probe sets. In comparing the discovered models for probes sets with the gene-averaged models (Table 2 column 3), there is remarkable agreement in terms of the genes (probe sets) added to the models, direction of effects, and total explained variance (R^2^). Thus we conclude that the models built here are robust to platform used for gene expression profiling, with similar models discovered regardless of platform or gene expression summarization method.

### Evaluation of Survival Prediction Model for Proneural Subtype in Validation Data Set

**Figure 3:**
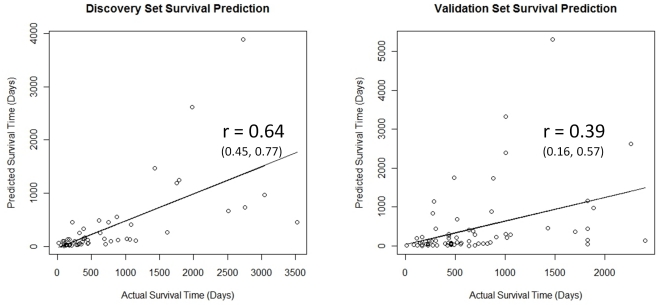
Graph of predicted and actual survival times in Proneural subtype for the discovery data set (left) and the validation data set (right) using the age-adjusted stepwise selection model (up to IFI44). The correlation between predicted and actual survival values is 0.64 (0.45, 0.77) in the discovery set and 0.39 (0.16, 0.57) in validation set.

In order to test the predictive ability of our discovered models in an independent data set not used for model building, we generated predicted survival times in a validation data set using the model built using stepwise regression in the Proneural subtype, including genes up to IFI44 (i.e. age, MX1, IFIT1, and IFI44). We chose this model because USP18 (the gene added after IFI44) is not present in the validation set, and we wanted the models between prediction and validation to be comparable. Plots of predicted versus actual survival time for both the discovery and validation data sets are shown in Figure 3. It can be seen from this figure that the predictive value of the multi-gene model in the Proneural subtype is retained in the validation set, with a correlation between predicted and actual survival times of 0.64 (95% confidence interval of 0.45, 0.77) in the discovery set and 0.39 (95% confidence interval of 0.16, 0.57) in the validation set. Thus although the prediction is a bit lower in the validation than the training set, as usually occurs when moving from training to testing sets, the 95% confidence interval for the correlation is greater than zero for both. If prediction models with just age are built, then a correlation between predicted and actual survival times of 0.45 (95% confidence interval of 0.21, 0.64) and 0.30 (95% confidence interval of 0.07, 0.51) are obtained in the discovery and validation sets, respectively, and thus higher predictive accuracy is achieved using the discovered gene expression model for prediction. Table S8 shows the results of directly fitting the discovered Cox model for the discovery Proneural sample in the validation Proneural sample (and also including terms to allow for potential study-specific survival rates in the four different studies included in the validation sample). The fitted model results are shown as hazard ratios and p-values for all of the parameters as well as total explained variance (R^2^) for the full model (28.2%) and a reduced model without the gene expression variables (20.3%).

### Network Analyses and Biological Interpretation of STAT1/IFN Gene Signature

**Figure 4:**
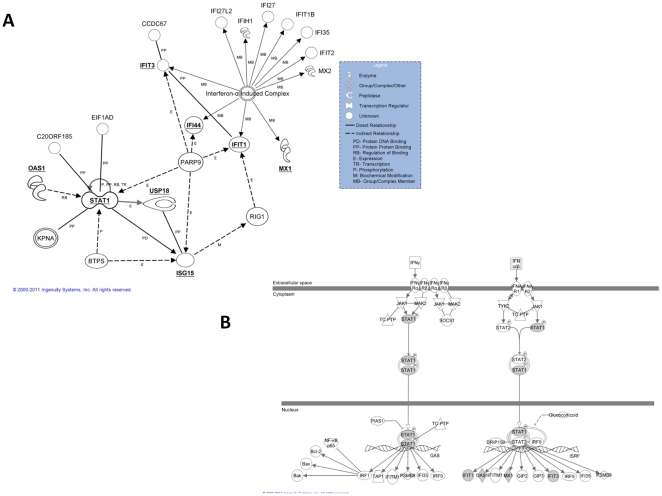
Functional Annotation networks from IPA (Ingenuity Pathway Analysis) that show documented gene relationships among the genes in the hypothesized eight gene STAT1/IFN gene set (A) and that show the functional relationships among these genes as they relate to Interferon signaling (B) (genes in eight gene signature are shaded).

**Figure 5:**
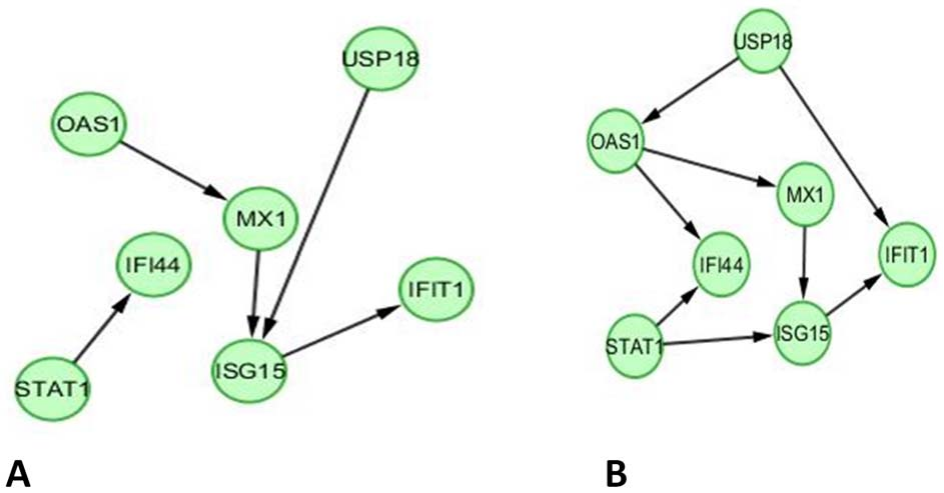
Discovered Bayesian Network for the full set of GBM (A) and the Proneural subtype (B). Growth-shrink algorithm was used and 80% bootstrap support for presence of edges and 50% bootstrap support for edge direction were required for the full sample (A), and 50% each were required for the Proneural subtype (B). With the more stringent criteria for the Proneural subtype (80% bootstrap support for edge presence) the OAS1->MX1 and ISG15->IFIT1 edges were discovered.

In order to provide biological context and interpretability of the STAT1/IFN gene signature, functional annotation networks were produced using IPA (Ingenuity Pathway Analysis, see Methods section) for the eight gene signature. Figure 4A illustrates the set of known functional relationships among the eight genes in the STAT1/IFN gene set and Figure 4B shows how these genes interact in the Interferon signaling cascade. The genes in the gene signature are bold-faced and underlined in 4A and bold-faced and shaded in 4B. In 4A, additional genes (a maximum of ten) are added by IPA to make connections among query (signature) genes separated by at most one gene. The IPA networks show most of these genes to be downstream targets of the Interferon/STAT1 signaling cascade, and indeed MX1, IFI44, ISG15, OAS1, and STAT1, which are predicted to be up-regulated with increased Interferon signaling, are shown to have significantly increased hazard ratios in the single gene analysis (Figure 1 and Table 1). Interestingly, the two genes not found to be significant in the single gene analysis, USP18 and IFIT1, are found to be significant in the multi-gene analysis for Proneurals (Table 2), with effects in the opposite direction (higher expression is protective, after correcting for the MX1 main effect). USP18 is a ubiquitin specific peptidase which specifically processes ISG15 (shown with the protein-protein interaction in Figure 4). USP18 has been demonstrated to be a regulator of susceptibility for interferon signaling and drug-induced apoptosis [10], and also to regulate EGFR-related expression and cancer cell survival [11], and thus may serve as an important counterbalance in this molecular signature. Interestingly, in the Bayesian Networks discovered for the full and Proneural data sets, shown in Figure 5 (A and B, respectively), the USP18-ISG15 interaction and the STAT1-ISG15 interactions are discovered, which lends support to the role of USP18 as a modulator of STAT1 signaling. Indeed these interactions are documented to exist as listed in Figure 4A (STAT1 -> ISG15 is a known protein-DNA interaction, and USP18 – ISG15 is a known protein-protein interaction). The Bayesian Networks also show connections between USP18 and IFIT1 (either directly in the full network or indirectly through ISG15 in the Proneural network), which may provide an explanation for why IFIT1 has a negative (protective) effect on survival in the Proneural subtype, as does USP18, after taking into account the MX1 main effect (see Table 2). Another general observation in comparing the two discovered networks in Figure 5 is that many of the edges overlap, suggesting that the Proneural subtype mechanisms dominate the behavior of the full sample.

## Discussion

Survival models for GBM patients from the Cancer Genome Atlas Project (TCGA) have been constructed using a hypothesized gene expression signature shown in previous experimental cell line studies to predict for radiation and chemotherapy resistance in tumor cells. Survival models were built for all GBM patients and in subtype-specific analyses, with and without age as a covariate, using single gene and multi-gene models. Our working hypothesis that over-expression of this gene signature predicts poor survival outcome in GBM patients was confirmed. In addition, it was demonstrated that the dependence of survival outcome on IFN/STAT1 gene expression was highly subtype-dependent, with strong dependence in the Proneural subtype and no detected dependence in the Classical and Mesenchymal subtypes. Additionally, the gene expression signature was shown to be almost invariant with age in predicting survival outcome, with little change in the signature in models with and without age. The multi-gene model constructed in the discovery set for the Proneural subtype was confirmed in an independent validation data set with a correlation between predicted and actual survival time of 0.39.

The dependency on the Proneural subtype for the IFN/STAT1 gene set highlights the context specificity for this signaling cascade and helps explain the apparent paradoxical downstream effects that IFN signaling promotes. In many model systems, IFN/STAT1 signaling promotes anti-proliferation and pro-apoptosis predominantly through the transcriptional modulation of key components of growth and apoptosis signaling including IRF1, Fas, FasL, TRAIL, p21waf1, and caspase-2, -3, and -7 [12], [13]. For this reason, it has been traditionally thought that STAT1 may function as a tumor suppressor. However, several reports have demonstrated that prolonged IFN signaling or constitutive STAT1 signaling promotes not only tumor growth, but also resistance to chemotherapy and radiation [4], [5], [7], [8], [14]. Given that the standard of care for patients following surgical resection and histopathologic diagnosis of GBM is involved field external beam radiotherapy alone or concurrent with temozolomide chemotherapy [15], [16] the overall survival data for patients whose pretreatment gene profiling was employed in the discovery and validation analyses described here is likely consistent with the predictive importance of these findings.

It has been hypothesized that constitutive activation of STAT1 promotes a “switch” from a cytotoxic signaling pathway to a pro-tumor survival phenotype. We suspect that this switch may be occurring in the Proneural subtype of GBM because it is this subtype that is thought to be the predominant secondary GBM subtype [9] that arises by progressive transformation from a lower to a higher grade glioma. It is possible that the more chronic natural history of the Proneural subtype yields a context in which constitutive IFN/STAT1 signaling generates the chemotherapy/radiation resistant phenotype. Indeed, only the Proneural subtype of GBM showed no improvement in outcome with intensive adjuvant therapy [9]. Therefore, targeting STAT1 for this molecular subtype may reverse this resistance to chemoradiation. A novel therapeutic approach for patients with GBM that has recently gained favor is the use of oncolytic viruses [17], [18], reviewed in [19]. Although the outcomes from multiple clinical trials have been less than optimal, there have been several reports of long-term (>5 yrs) clinical remissions in an otherwise unclassified cohort of patients. Whether or not GBM patients who display an elevation in IFN/STAT1-mediated gene activation have a heightened resistance to virus-mediated infection and oncolysis remains to be determined, but if this proves to be so, it could form a rational basis for prescreening GBM patients for this therapy. Speaking more generally, we are optimistic that the IFN/STAT1 mechanism shown in this report to be related to disease progression in Proneurals could be exploited in future research for either tailored treatment selection in this subgroup or for rational design of novel therapeutic agents.

## Materials and Methods

### Data

Clinical and gene expression data was obtained from the Cancer Genome Atlas Project. Gene expression data from three separate microarray platforms was summarized by gene using a method described elsewhere [9]. In this list of genes one was missing from the 8-gene set (IFIT3). The validation data set was derived from four separate studies as described in [9]. The summarized clinical data for the validation set was kindly provided by Neil Hayes. In addition, gene expression data for individual probe sets for the Affymetrix and Agilent platforms were analyzed as well; these data were obtained through the TCGA data portal. Of the 202 samples in the original data set, 2 were non-GBM, and eight were removed for having prior glioma, leaving 192 individuals containing data for the gene-summarized gene expression and clinical characteristics, and 191 individuals containing gene expression data in the Affymetrix and Agilent platforms. Filtering out prior glioma was performed to be consistent with the original goals of the TCGA to characterize primary GBMs. 246 individuals were included from the validation data set. More detailed clinical characteristics of the both samples are contained in [9]. In addition, the number of patients by subtype are given in Table S9 for discovery and validation sets. The assignment of subtype is done using a gene expression-based classification algorithm in [8].

### Statistical and Network Analysis

Single gene Cox proportional hazards models were fit using Proc PHReg in SAS version 9.2 with predicted survival curves generated using the median age and the 1^st^ and 3^rd^ quartiles of gene expression for each gene. In particular, the survival curves were predicted based on exponentiating Breslow's baseline cumulative hazard rate at a median value of age. The parameter estimates for the gene categories used in the exponentiation have been estimated in the presence of the effect of age. For building multi-gene models, forward stepwise regression using the Cox proportional hazards model was performed using the coxph routine in the Survival package in R version 2.10.1. The step function in the R stats package was used to do forward selection with the stopping criteria being a non-decreasing Akaike Information Criteria (AIC) value. Model R^2^ was calculated to account for censoring using a formula given in [20]. Violation of the proportional hazards assumption was tested using the cox.zph function which computes a test for each model parameter and a global model test using a method described in [21].

Penalized survival analysis using Elastic Net was performed using the glmnet package in R. For generating predicted survival times from the discovered multi-genic Cox models in both the discovery and validation sets, a procedure described in [22] was used after designating the exponential parametric form. The value of the tuning parameter for the Elastic Net method is selected such that mean cross-validation error is minimized. This was implemented using the function cv.glmnet available in the package glmnet.

Functional annotation networks were generated using Ingenuity Pathway Analysis or IPA (www.ingenuity.com), which provides a graphical representation of the molecular relationships between genes. The network was generated initially using the 8 gene set, and was expanded with a maximum of 10 genes that are connected to the initial genes. Molecules are represented as nodes, and the biological relationship between two nodes is represented as an edge (line). All edges are supported by at least 1 reference from the literature, from a textbook, or from canonical information stored in the Ingenuity Pathways Knowledge Base. Direct relationships are indicated by solid lines and indirect through dashed lines. Line beginnings and endings illustrate the direction of the relationship (e.g. arrow head indicates gene A influences gene B). Nodes are displayed using various shapes that represent the functional class of the gene product. Edges are displayed with various labels that describe the nature of the relationship between the nodes (e.g., P for phosphorylation, T for transcription).

Bayesian Networks were discovered using the growth-shrink algorithm implemented in the package BNLearn version 1.9 in R with a custom routine for finding bootstrap support for edge presence and direction.

## Supporting Information

Figure S1 Survival Curves for age-adjusted Cox Proportional Hazard predicted survival for 1^st^ quartile (red) and 3^rd^ quartile (blue) gene expression values for each gene in the full data set.
(TIF)

Table S1 Single Gene Cox Proportional Hazards Models with age adjustment for seven genes available in the TCGA discovery (gene-averaged) data set for Classical, Mesenchymal, and Neural subtypes. Estimated hazard ratio and p-values are given for each gene.
(DOC)

Table S2 Correlation of gene expression values (lower diagonal is the correlation, upper diagonal is the p-value for the test of zero correlation) for the genes in the full TCGA data set.
(DOC)

Table S3 Correlation of gene expression values (lower diagonal is the correlation, upper diagonal is the p-value for the test of zero correlation) for the genes in the Proneural subtype of the TCGA data set.
(DOC)

Table S4 Test of proportional hazards violation for individual model terms and global model test for single gene Cox models in Full and Proneural data sets.
(DOC)

Table S5 Test of proportional hazards violation for individual model terms and global model test for stepwise multi-gene Cox models in Full and Proneural data sets.
(DOC)

Table S6 Cox Proportional Hazard model hazard ratios and model R^2^ for expression models built with age and individual Affymetrix probe set variables using Elastic net regularization.
(DOC)

Table S7 Cox Proportional Hazard model hazard ratios and model R^2^ for expression models built with age and individual Agilent probeset variables using Elastic net regularization. Note the following probe sets in the analysis but were not selected in any of the models and thus not included in the table: for OAS1 (A_23_P64828, A_24_P253162, and NM_016816_1_1099), for ISG15 (A_23_P811, A_23_P815, A_23_P819, A_23_P404628, A_32_P99533, A_32_P99534, NM_005101_1_144, and NM_005101_1_275), and for IFIT3 (A_23_P35404, A_23_P35405, and A_23_P35412).
(DOC)

Table S8 Cox Proportional Hazard model for the Proneural sample in the validation set using the stepwise selection variables found for the Proneural sample in the discovery set (with the exception of USP18 which is not present in the gene-averaged validation gene expression sample, see Table 2), and including a factor variable for study center to allow for study-specific survival rates. Estimated hazard ratios and p values are shown for each term in the model, and the full model R^2^ as well as the reduced R^2^ when only including age and center variables are shown.
(DOC)

Table S9 Patient Characteristics in discovery (TCGA) and validation data sets. For the discovery set, only GBM samples without prior glioma were used.
(DOC)
