# Supplementary figures and images for: Expression Signature of IFN/STAT1 Signaling Genes Predicts Poor Survival Outcome in Glioblastoma Multiforme in a Subtype-Specific Manner
Source: PLoS One. 2012 Jan 5;7(1):e29653. doi: 10.1371/journal.pone.0029653 (PMC3252343; doi:10.1371/journal.pone.0029653)

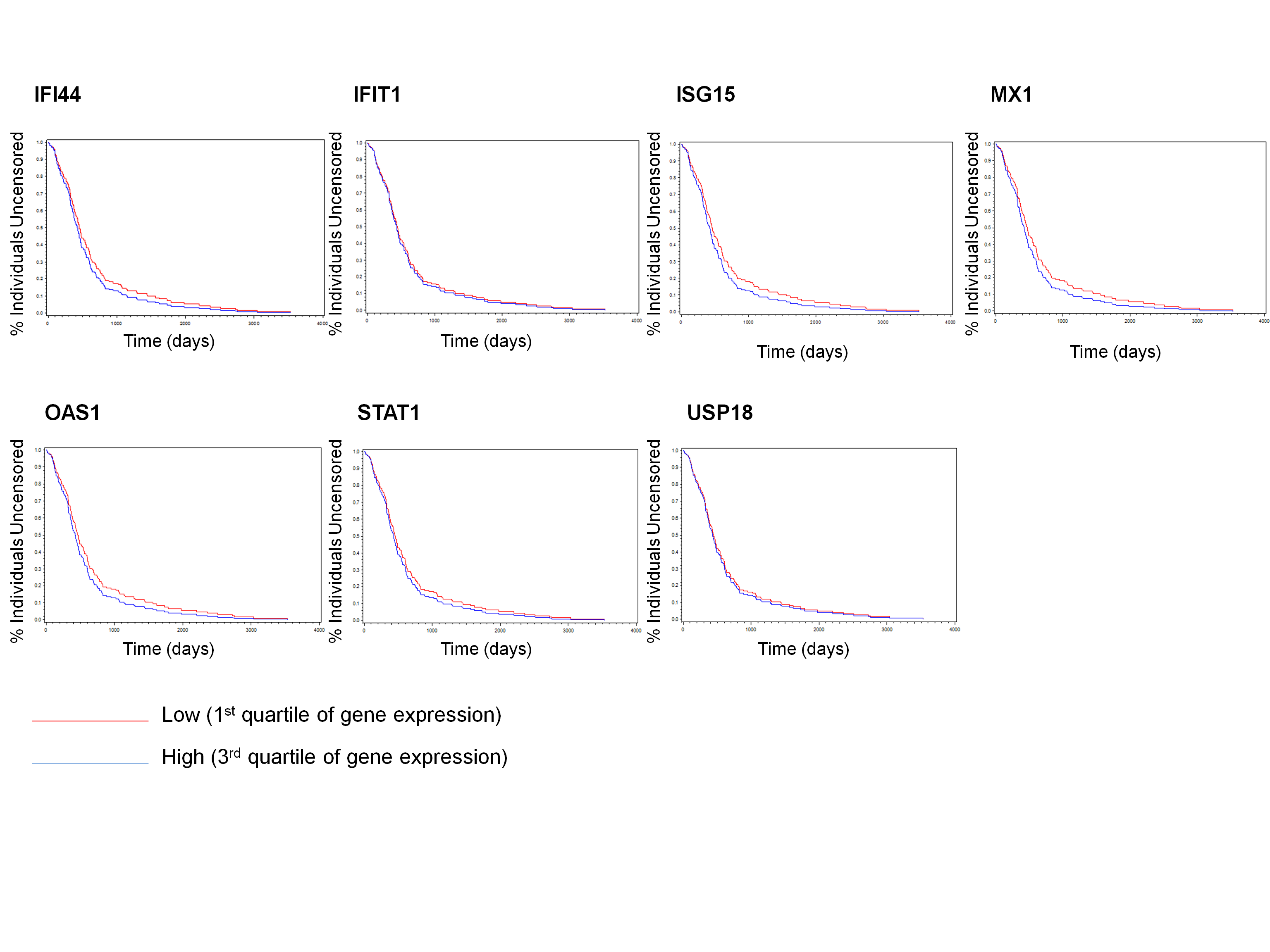

Supplement: Figure S1 — Survival Curves for age-adjusted Cox Proportional Hazard predicted survival for 1st quartile (red) and 3rd quartile (blue) gene expression values for each gene in the full data set. (TIF) [file pone.0029653.s001.tif]
