# Supplementary material for: Expression Signature of IFN/STAT1 Signaling Genes Predicts Poor Survival Outcome in Glioblastoma Multiforme in a Subtype-Specific Manner
Source: PLoS One. 2012 Jan 5;7(1):e29653. doi: 10.1371/journal.pone.0029653 (PMC3252343; doi:10.1371/journal.pone.0029653)
Supplement: Table S1 — Single Gene Cox Proportional Hazards Models with age adjustment for seven genes available in the TCGA discovery (gene-averaged) data set for Classical, Mesenchymal, and Neural subtypes. Estimated hazard ratio and p-values are given for each gene. (DOC) [file pone.0029653.s002.doc]

| ***Gene*** | ***Classical*** |  | ***Mesenchymal*** |  | ***Neural*** |  |
| --- | --- | --- | --- | --- | --- | --- |
|  | *HR* | *p value* | *HR* | *p value* | *HR* | *p value* |
| **IFI44** | 1.02 | 0.885 | 0.95 | 0.830 | 1.71 | 0.051 |
| **IFIT1** | 0.97 | 0.769 | 1.07 | 0.579 | 1.44 | 0.053 |
| **ISG15** | 0.98 | 0.843 | 1.07 | 0.687 | 1.44 | 0.081 |
| **MX1** | 0.99 | 0.904 | 1.07 | 0.641 | 1.23 | 0.309 |
| **OAS1** | 0.94 | 0.669 | 1.16 | 0.493 | 1.58 | 0.061 |
| **STAT1** | 0.95 | 0.734 | 1.03 | 0.919 | 1.48 | 0.276 |
| **USP18** | 0.92 | 0.659 | 1.05 | 0.803 | **1.76** | **0.020** |
