# Supplementary material for: Expression Signature of IFN/STAT1 Signaling Genes Predicts Poor Survival Outcome in Glioblastoma Multiforme in a Subtype-Specific Manner
Source: PLoS One. 2012 Jan 5;7(1):e29653. doi: 10.1371/journal.pone.0029653 (PMC3252343; doi:10.1371/journal.pone.0029653)
Supplement: Table S2 — Correlation of gene expression values (lower diagonal is the correlation, upper diagonal is the p-value for the test of zero correlation) for the genes in the full TCGA data set. (DOC) [file pone.0029653.s003.doc]

| **Corr/P-value** | **IFI44** | **IFIT1** | **ISG15** | **MX1** | **OAS1** | **STAT1** | **USP18** |
| --- | --- | --- | --- | --- | --- | --- | --- |
| **IFI44** | 1 | <.0001 | <.0001 | <.0001 | <.0001 | <.0001 | <.0001 |
| **IFIT1** | 0.71749 | 1 | <.0001 | <.0001 | <.0001 | <.0001 | <.0001 |
| **ISG15** | 0.80143 | 0.85899 | 1 | <.0001 | <.0001 | <.0001 | <.0001 |
| **MX1** | 0.82434 | 0.75478 | 0.8517 | 1 | <.0001 | <.0001 | <.0001 |
| **OAS1** | 0.77343 | 0.71441 | 0.77483 | 0.83725 | 1 | <.0001 | <.0001 |
| **STAT1** | 0.78311 | 0.67074 | 0.74886 | 0.76027 | 0.73293 | 1 | <.0001 |
| **USP18** | 0.66698 | 0.71984 | 0.75445 | 0.6775 | 0.6677 | 0.66471 | 1 |
