# Supplementary material for: Expression Signature of IFN/STAT1 Signaling Genes Predicts Poor Survival Outcome in Glioblastoma Multiforme in a Subtype-Specific Manner
Source: PLoS One. 2012 Jan 5;7(1):e29653. doi: 10.1371/journal.pone.0029653 (PMC3252343; doi:10.1371/journal.pone.0029653)
Supplement: Table S3 — Correlation of gene expression values (lower diagonal is the correlation, upper diagonal is the p-value for the test of zero correlation) for the genes in the Proneural subtype of the TCGA data set. (DOC) [file pone.0029653.s004.doc]

| **Corr/P-value** | **IFI44** | **IFIT1** | **ISG15** | **MX1** | **OAS1** | **STAT1** | **USP18** |
| --- | --- | --- | --- | --- | --- | --- | --- |
| **IFI44** | 1 | <.0001 | <.0001 | <.0001 | <.0001 | <.0001 | <.0001 |
| **IFIT1** | 0.66717 | 1 | <.0001 | <.0001 | <.0001 | <.0001 | <.0001 |
| **ISG15** | 0.82799 | 0.77944 | 1 | <.0001 | <.0001 | <.0001 | <.0001 |
| **MX1** | 0.76212 | 0.65253 | 0.83463 | 1 | <.0001 | <.0001 | <.0001 |
| **OAS1** | 0.83304 | 0.70317 | 0.84481 | 0.85029 | 1 | <.0001 | <.0001 |
| **STAT1** | 0.73846 | 0.65022 | 0.78117 | 0.69719 | 0.73514 | 1 | <.0001 |
| **USP18** | 0.57143 | 0.71039 | 0.66385 | 0.51537 | 0.65361 | 0.63045 | 1 |
