# Supplementary material for: Expression Signature of IFN/STAT1 Signaling Genes Predicts Poor Survival Outcome in Glioblastoma Multiforme in a Subtype-Specific Manner
Source: PLoS One. 2012 Jan 5;7(1):e29653. doi: 10.1371/journal.pone.0029653 (PMC3252343; doi:10.1371/journal.pone.0029653)
Supplement: Table S4 — Test of proportional hazards violation for individual model terms and global model test for single gene Cox models in Full and Proneural data sets. (DOC) [file pone.0029653.s005.doc]

| **Data Set** | **Term** | **Rho** | **ChiSq** | **p value** |
| --- | --- | --- | --- | --- |
| Full | age | -0.198 | 7.75 | 0.00539 |
|  | IFI44 | 0.100 | 1.77 | 0.18391 |
|  | GLOBAL |  | 9.25 | 0.00982 |
|  | age | -0.195 | 7.4717 | 0.00627 |
|  | IFIT1 | 0.022 | 0.0959 | 0.75685 |
|  | GLOBAL |  | 7.7979 | 0.02026 |
|  | age | -0.189 | 7.12 | 0.0076 |
|  | ISG15 | 0.140 | 3.39 | 0.0654 |
|  | GLOBAL |  | 11.09 | 0.0039 |
|  | age | -0.204 | 8.43 | 0.00368 |
|  | MX1 | 0.121 | 2.46 | 0.11677 |
|  | GLOBAL |  | 10.57 | 0.00507 |
|  | age | -0.198 | 7.75 | 0.00539 |
|  | IFI44 | 0.100 | 1.77 | 0.18391 |
|  | GLOBAL |  | 9.25 | 0.00982 |
|  | age | -0.202 | 8.21 | 0.00417 |
|  | OAS1 | 0.128 | 2.9 | 0.0888 |
|  | GLOBAL |  | 10.85 | 0.00441 |
|  | age | -0.198 | 7.776 | 0.00529 |
|  | STAT1 | 0.061 | 0.616 | 0.43239 |
|  | GLOBAL |  | 8.405 | 0.01496 |
|  | age | -0.185 | 6.78 | 0.00924 |
|  | USP18 | 0.076 | 1.21 | 0.27156 |
|  | GLOBAL |  | 8.74 | 0.01266 |
| Proneural | age | -0.387 | 6.48 | 0.0109 |
|  | IFI44 | 0.086 | 0.26 | 0.6112 |
|  | GLOBAL |  | 7.48 | 0.0237 |
|  | age | -0.486 | 10.25 | 0.0014 |
|  | IFIT1 | 0.037 | 0.07 | 0.7896 |
|  | GLOBAL |  | 10.32 | 0.0057 |
|  | age | -0.398 | 7.58 | 0.0059 |
|  | ISG15 | 0.068 | 0.21 | 0.6510 |
|  | GLOBAL |  | 9.15 | 0.0103 |
|  | age | -0.415 | 9.22 | 0.0024 |
|  | MX1 | -0.057 | 0.14 | 0.7068 |
|  | GLOBAL |  | 9.55 | 0.0085 |
|  | age | -0.387 | 6.48 | 0.0109 |
|  | IFI44 | 0.086 | 0.26 | 0.6112 |
|  | GLOBAL |  | 7.48 | 0.0237 |
|  | age | -0.377 | 6.90 | 0.0086 |
|  | OAS1 | 0.144 | 1.04 | 0.3076 |
|  | GLOBAL |  | 9.63 | 0.0081 |
|  | age | -0.400 | 7.78 | 0.0053 |
|  | STAT1 | -0.037 | 0.05 | 0.8194 |
|  | GLOBAL |  | 8.24 | 0.0162 |
|  | age | -0.445 | 9.22 | 0.0024 |
|  | USP18 | 0.031 | 0.06 | 0.8085 |
|  | GLOBAL |  | 10.19 | 0.0061 |
