# Supplementary material for: Expression Signature of IFN/STAT1 Signaling Genes Predicts Poor Survival Outcome in Glioblastoma Multiforme in a Subtype-Specific Manner
Source: PLoS One. 2012 Jan 5;7(1):e29653. doi: 10.1371/journal.pone.0029653 (PMC3252343; doi:10.1371/journal.pone.0029653)
Supplement: Table S5 — Test of proportional hazards violation for individual model terms and global model test for stepwise multi-gene Cox models in Full and Proneural data sets. (DOC) [file pone.0029653.s006.doc]

| **Subset** | **Model** | **Term** | **Rho** | **ChiSq** | **p value** |
| --- | --- | --- | --- | --- | --- |
| Full | SW with age | age | -0.208 | 8.97 | 0.0027 |
|  |  | MX1 | 0.139 | 2.59 | 0.1078 |
|  |  | IFIT1 | -0.081 | 0.99 | 0.3193 |
|  |  | GLOBAL |  | 10.62 | 0.0140 |
| Full | SW no age | MX1 | 0.072 | 0.68 | 0.4090 |
|  |  | IFIT1 | -0.006 | 0.01 | 0.9408 |
|  |  | GLOBAL |  | 1.40 | 0.4970 |
| Proneural | SW with age | age | -0.309 | 6.03 | 0.0141 |
|  |  | MX1 | -0.119 | 0.89 | 0.3466 |
|  |  | IFIT1 | 0.024 | 0.04 | 0.8421 |
|  |  | IFI44 | 0.274 | 4.01 | 0.0453 |
|  |  | USP18 | -0.180 | 1.49 | 0.2216 |
|  |  | ISG15 | -0.010 | 0.01 | 0.9276 |
|  |  | GLOBAL |  | 10.30 | 0.1125 |
| Proneural | SW no age | MX1 | -0.036 | 0.05 | 0.8256 |
|  |  | IFIT1 | -0.044 | 0.08 | 0.7728 |
|  |  | IFI44 | 0.164 | 1.37 | 0.2421 |
|  |  | USP18 | -0.090 | 0.32 | 0.5738 |
|  |  | GLOBAL |  | 1.44 | 0.8365 |
