# Supplementary material for: Expression Signature of IFN/STAT1 Signaling Genes Predicts Poor Survival Outcome in Glioblastoma Multiforme in a Subtype-Specific Manner
Source: PLoS One. 2012 Jan 5;7(1):e29653. doi: 10.1371/journal.pone.0029653 (PMC3252343; doi:10.1371/journal.pone.0029653)
Supplement: Table S6 — Cox Proportional Hazard model hazard ratios and model R2 for expression models built with age and individual Affymetrix probe set variables using Elastic net regularization. (DOC) [file pone.0029653.s007.doc]

|  | **Probe Set** | **All** | **Proneural** | **Neural** | **Classical** | **Mesenchymal** |
| --- | --- | --- | --- | --- | --- | --- |
| Age |  | 1.02 | 1.03 | 1.03 |  | 1.02 |
| MX1 | 202086_at | 1.03 | 1.48 |  |  |  |
| IFIT1 | 203153_at |  | 0.72 | 1.04 |  |  |
| USP18 | 219211_at |  | 0.93 | 1.08 |  |  |
| IFI44 | 214059_at |  | 1.13 |  |  |  |
|  | 214453_s_at | 1.01 | 1.20 | 1.20 |  |  |
| OAS1 | 202869_at |  | 1.04 | 1.06 |  |  |
|  | 205552_s_at |  |  |  |  |  |
| ISG15 | 205483_s_at |  |  |  |  |  |
| STAT1 | 200887_s_at |  | 1.34 | 1.06 |  |  |
|  | 209969_s_at |  | 1.10 |  |  |  |
|  | AFFX-HUMISGF3A/M97935_3_at |  |  |  |  |  |
|  | AFFX-HUMISGF3A/M97935_5_at |  |  |  |  |  |
|  | AFFX-HUMISGF3A/M97935_MA_at |  | 0.78 |  |  |  |
|  | AFFX-HUMISGF3A/M97935_MB_at |  |  |  |  |  |
| IFIT3 | 204747_at |  |  |  |  |  |
| **R^2^** |  | **31%** | **80%** | **44%** | **0%** | **22%** |
