# Supplementary material for: Expression Signature of IFN/STAT1 Signaling Genes Predicts Poor Survival Outcome in Glioblastoma Multiforme in a Subtype-Specific Manner
Source: PLoS One. 2012 Jan 5;7(1):e29653. doi: 10.1371/journal.pone.0029653 (PMC3252343; doi:10.1371/journal.pone.0029653)
Supplement: Table S7 — Cox Proportional Hazard model hazard ratios and model R2 for expression models built with age and individual Agilent probeset variables using Elastic net regularization. Note the following probe sets in the analysis but were not selected in any of the models and thus not included in the table: for OAS1 (A_23_P64828, A_24_P253162, and NM_016816_1_1099), for ISG15 (A_23_P811, A_23_P815, A_23_P819, A_23_P404628, A_32_P99533, A_32_P99534, NM_005101_1_144, and NM_005101_1_275), and for IFIT3 (A_23_P35404, A_23_P35405, and A_23_P35412). (DOC) [file pone.0029653.s008.doc]

|  | **Probe Set** | **All** | **Proneural** | **Neural** | **Classical** | **Mesenchymal** |
| --- | --- | --- | --- | --- | --- | --- |
| Age |  | 1.02 | 1.03 | 1.02 |  | 1.02 |
| MX1 | A_23_P17663 | 1.03 | 1.14 |  |  |  |
|  | A_23_P17664 |  | 1.15 |  |  |  |
|  | NM_002462_2_2647 |  | 1.20 |  |  |  |
|  | NM_002462_2_2693 | 1.05 | 1.21 |  |  |  |
| IFIT1 | A_23_P52266 |  | 0.87 |  |  |  |
|  | A_23_P52267 |  | 0.87 |  |  |  |
| USP18 | A_23_P132159 |  | 0.85 | 1.17 |  |  |
|  | A_23_P132162 |  |  | 1.03 |  |  |
|  | A_32_P132206 |  |  | 1.09 |  |  |
|  | A_32_P132207 |  | 0.92 | 1.11 |  |  |
| IFI44 | A_23_P23073 |  | 1.12 |  |  |  |
|  | A_23_P23074 |  | 1.14 |  |  |  |
| STAT1 | A_23_P56630 |  |  | 1.07 |  |  |
|  | A_24_P274270 |  |  |  |  |  |
|  | A_23_P303721 |  |  |  |  |  |
|  | NM_007315_2_4047 |  |  |  |  |  |
| **R^2^** |  | **30%** | **78%** | **46%** | **0%** | **21%** |
