# Supplementary material for: Expression Signature of IFN/STAT1 Signaling Genes Predicts Poor Survival Outcome in Glioblastoma Multiforme in a Subtype-Specific Manner
Source: PLoS One. 2012 Jan 5;7(1):e29653. doi: 10.1371/journal.pone.0029653 (PMC3252343; doi:10.1371/journal.pone.0029653)
Supplement: Table S8 — Cox Proportional Hazard model for the Proneural sample in the validation set using the stepwise selection variables found for the Proneural sample in the discovery set (with the exception of USP18 which is not present in the gene-averaged validation gene expression sample, see Table 2), and including a factor variable for study center to allow for study-specific survival rates. Estimated hazard ratios and p values are shown for each term in the model, and the full model R2 as well as the reduced R2 when only including age and center variables are shown. (DOC) [file pone.0029653.s009.doc]

|  | **HR** | **p value** |
| --- | --- | --- |
| Age | 1.03 | 0.0086 |
| MX1 | 1.62 | 0.0220 |
| IFIT1 | 0.78 | 0.1100 |
| IFI44 | 0.92 | 0.7400 |
| Murat | 0.47 | 0.1200 |
| Phillips | 0.67 | 0.3400 |
| Rembrandt/Fine | 0.46 | 0.0890 |
| ***R^2^*** | *Full* | 28.2% |
| ***R^2^*** | *No Genes* | 20.3% |
