# Supplementary material for: Expression Signature of IFN/STAT1 Signaling Genes Predicts Poor Survival Outcome in Glioblastoma Multiforme in a Subtype-Specific Manner
Source: PLoS One. 2012 Jan 5;7(1):e29653. doi: 10.1371/journal.pone.0029653 (PMC3252343; doi:10.1371/journal.pone.0029653)
Supplement: Table S9 — Patient Characteristics in discovery (TCGA) and validation data sets. For the discovery set, only GBM samples without prior glioma were used. (DOC) [file pone.0029653.s010.doc]

|  | **Proneural** | **Neural** | **Classical** | **Mesenchymal** | **Total** |
| --- | --- | --- | --- | --- | --- |
| **Discovery (TCGA)** | 55 | 27 | 53 | 57 | 192 |
| **Validation** | 69 | 40 | 63 | 74 | 246 |
